# Current News

**Published:** 1905-06

**Authors:** 


### Current News.

### LEWIS AND CLARK DENTAL CONGRESS.

*To the Members of the National Dental Association:*

Gentlemen,—The Executive Committee of the Lewis and Clark Dental Congress invites the National Dental Association to change its meeting place for this year to Portland, Ore., and its dates to correspond with those of the Congress, combining the two meetings and lengthening, if necessary, the sessions to six days,—July 17 to 22.

If this action is not possible, your attention is called to the opportunity of attending both meetings,—the Lewis and Clark Dental Congress closing July 20, in ample time to allow those who desire to reach Buffalo for the opening day of the National Association.

The dentists of the West are taking a great interest in the Congress, and it is demonstrated that more than one thousand from this section alone will attend.

This fact, together with the class and size of the programme which is being formed, the conveniences of the meeting hall, the interest being displayed by the large manufacturers who will exhibit their goods, and many other things as showing the result of the business-like methods adopted in conducting the work of the Congress, as well as the opportunity offered for an enjoyable and instructive vacation trip to the great West, at rates lower than ever before quoted,—one-half fare or less from all sections of the United States,—warrant the committee in asking that the courtesy of attendance and participation in the Congress be granted by the members of the National Dental Association and dentists generally residing in the Eastern States.

You are assured that this will be appreciated, and you may be confident that if you will attend you will return to your homes after the meeting with a feeling of gratification that you made the trip, saw the most magnificent scenery in the United States, and spent the month of July in a most delightful climate.

All of this will have been accomplished for but a small portion of the expense incident to such a trip at any time in the past, and for an indefinite period in the future.

Membership in the Congress, with or without attendance, will entitle each one to a bound copy of the proceedings.

Will you indicate your intentions concerning the Congress, and if you will participate correspond with the Chairman of Committees?

ARTHUR W. CHANCE,
*Secretary.*

WHERE THE CONGRESS WILL BE HELD.

The Armory of the Oregon National Guard will be the meeting hall of the Lewis and Clark Dental Congress. It is the largest armory west of Chicago and St. Louis. It is 200 x 200 feet, first story of stone, second of brick, provided with all modern conveniences.

The location is very central—eight squares or blocks from all large hotels, post-office, railroad terminal depot, and active centre of city. (City squares or blocks are two hundred feet in length.)

The Assembly hall, where the general meetings take place, is 60 x 100 and will accommodate one thousand people. The Exhibit hall, where the manufacturers' exhibits and professional clinics take place, is 100 x 200 feet, contains twenty thousand square feet of space, is located on the ground floor, and in the north end of the building, where the daylight facilities are first class.

HOTELS AND ROOMS.

Hotel Portland, European, $2.50 to $3.00 single, $5.00 to $6.00 double; Hotel Imperial, European, $2.00 to $4.00; Hotel Perkins, $2.00 to $4.00. Many other hotels at lower rates.

*Private Hotels.*—The Eaton, European, $1.50 to $5.00; The Norton, American, $4.00 to $6.00; Elton Court, $3.00 to $5.00 European, $5.00 to $7.00 American; Hobart-Curtis, $3.00 to $7.00 American.

EXPOSITION ACCOMMODATION BUREAU (INC.).

Under the supervision and control of Lewis and Clark Centennial Exposition and Oriental Fair five thousand rooms in private families convenient to the Armory have been secured which are offered at the following rates: $1.25 per day for single room; $2.50 per day for double room occupied by one or by two people. Business address: Goodnough Building, Fifth and Yamhill Streets.

Reservations for any of the above hotels or for rooms of the Accommodation Bureau, made in advance.

FEATURES OF THE PROGRAMME.

The essays at the Lewis and Clark Dental Congress will be by th. leading men in the dental and medical professions of the United States and Europe. Essayists are being selected with a view of making this part of the programme unique and original in character. Symposiums on various subjects will be given. There will be no sections in the meeting, all of the essays being delivered before the entire Congress.

During the four days that the Congress will be in session there will be one hundred and fifty professional clinics given by dentists from every part of the world. The clinical programme will be a large feature of the Congress, and clinicians have been secured to demonstrate every operation in the range of our profession, especially the newest and latest methods in both operative and prosthetic work. The clinical programme is receiving additions daily.

There will be fifty or more exhibits, covering fifteen thousand square feet, comprising every variety of dental goods made, and there will be clinical demonstrations by manufacturers of every dental appliance and specialty on the market.

Railroad rates will be low,—$56.00 round trip from Chicago,

$45.00 from Missouri River, St. Paul, Minneapolis, Duluth, and the Superiors, one-half regular fare from territory in the scope of the Lewis and Clark Exposition.

In connection with above rates, Eastern lines have authorized one fare for the round trip from point of origin to Chicago and Missouri River.

The membership ticket, which entitles the holder to all privileges, will be five dollars ($5.00).

---

### NATIONAL DENTAL ASSOCIATION.

The following programmes will be offered for the consideration of Sections I. and II. in Buffalo, July 25 to 27, 1905:

### SECTION I.

"Orthodontia." Dr. Calvin S. Case, Chicago; Dr. C. Edmund Kells, Jr., New Orleans, La.; Dr. V. H. Jackson, New York; Dr. R. Ottolengui, New York.

"Prosthetic Dentistry." Dr. H. H. Johnson, Macon, Ga.; Frederic Freeman, Boston.

"Crown- and Bridge-Work." Dr. W. Storer Howe, Philadelphia.

"The D.D.S. Abroad." Special paper by Dr. R. H. Hofheinz, Rochester, N. Y.

Dr. Thomas P. Hinman, *Chairman,*
Atlanta, Ga.
Dr. J. G. Fife, *Secretary,*
Dallas, Tex.

### SECTION II.

"Gold as a Filling Material." J. V. Conzett, Dubuque, Iowa.

Lantern Lecture: "Pioneer Manipulators of Gold-Foil." B. L. Thorp, St. Louis, Mo.

"Dental Education." Chas. Milton Ford, New York City.

"The Necessity for a Method of preserving the Integrity of the Interproximal Space." W. R. Clack, Clear Lake, Iowa.

"A Few Experiments in Porcelain." Dr. D. O. M. LeCron, St. Louis, Mo.

"A Century of Standard Dental Writings." Dr. D. W. Fellows, Portland, Me.

"Operative Dentistry." Dr. B. Holly Smith, Baltimore, Md.

(To be announced.) Professor Geo. B. Snow, Buffalo, N. Y.; Dr. W. H. K. Moyer, Little Falls, Minn.

"Nomenclature." Dr. D. R. Stubblefield, Nashville, Tenn.

"The Nomenclature of Orthodontia." Dr. S. H. Guilford, Philadelphia.

DR. HOWARD E. ROBERTS, *Chairman*,
Philadelphia, Pa.
DR. C. S. BUTLER, *Secretary*,
Buffalo, N. Y.

---

### SOUTH DAKOTA STATE BOARD OF DENTAL EXAMINERS.

THE next meeting of the South Dakota State Board of Dental Examiners will be held at Mitchell, S. D., July 11, beginning at 1.30 P.M. All candidates will be required to do practical work in both operative and prosthetic dentistry, and should bring all instruments and materials necessary to do the same. Vulcanizer, lathe, and swaging appliances will be furnished by the board. Application, together with fee of ten dollars, must positively be in the hands of the Secretary before July 7.

G. W. COLLINS.

---

### "F. D. I." INTERNATIONAL DENTAL FEDERATION.

THE next annual meeting of the Executive Council of the Fédération Dentaire Internationale will convene in Hanover, Germany, August 7, 1905, immediately following the annual meeting of the Central-Verein Deutscher Zahnarzte. Announcement of the programme for the meeting and the projected work for the Federation during the present period will shortly be made through the dental journals and through the official bulletin of the Federation.

EDWARD C. KIRK,
*Secretary-General.*

